# Alkaline Material Effects on Roots of Teeth

**DOI:** 10.3390/ma10121412

**Published:** 2017-12-10

**Authors:** Sowmya Shetty, Sam L. Kahler, Bill Kahler

**Affiliations:** 1 School of Dentistry, The University of Queensland, Brisbane 4006, Australia; w.kahler@uq.edu.au; 2 School of Biomedical Sciences, The University of Queensland, Brisbane 4006, Australia; s4393079@student.uq.edu.au

**Keywords:** endodontic treatment, alkaline material, mechanical properties, tooth root, fracture

## Abstract

The aim of this review was to identify and analyse all studies related to the effects of alkaline materials used in dentistry on roots of teeth. The first part of the review focused on mechanical property alterations of root dentine due to sodium hypochlorite (SH) used as an irrigant solution based on MeSH (Medical Subject Heading) terms from a previous study by Pascon et al in 2009. The second part reviewed literature on calcium hydroxide (CH), mineral trioxide aggregate (MTA) and other alkaline materials used as root canal dressings or filling materials. Additional MeSH terms used included “compressive strength”, “elastic modulus” “flexural strength”, “fracture strength” and “fracture resistance”. The language filter was English. Of the initial 205 articles identified, 49 were included in this review, of which 29 were on SH, 21 on CH/MTA, and 1 relating to both. Many in vitro studies indicated a strong link between reduced mechanical properties of roots of teeth or radicular dentine treated with SH, and when sealers or root fillings with CH- or MTA-based materials were placed in contact with roots or radicular dentine. Recent literature indicates that the association between reduced mechanical properties and alkaline sealers and/or root-filling materials is not as straightforward as previously assumed, and requires further investigation using more valid experimental models.

## 1. Introduction

Alkaline materials such as sodium hypochlorite (SH), calcium hydroxide (CH) and mineral trioxide aggregate (MTA) have been used for various applications within dentistry. 

Sodium hypochlorite is widely used as an irrigant in endodontics due to its disinfection actions and its ability to dissolve necrotic organic material within the root canal system of teeth [1,2]. Dentine consists of a hydrated organic matrix which is mainly type 1 collagen, which comprises 22% by weight of the material, into which is embedded an inorganic phase of carbonated apatite that contributes to its mechanical properties [3]. SH is a non-specific proteolytic agent that also removes carbonate ions from dentine [4,5]. SH was shown to produce concentration-dependent collagen depletion leaving an unbound hydroxyapatite and an apatite-rich and collagen-sparse dentine subsurface [5]. Therefore, SH may affect the mechanical properties of dentine by damaging its organic matrix by making dentine more brittle. This topic was reviewed by Pascon et al. who concluded that SH alters the mechanical properties of dentine [6]. These authors systematically reviewed the effect of mechanical analysis, elastic modulus, hardness, roughness, compressive strength and the flexural strength of dentine. 

A recent paper was entitled ‘Primum non necere’ (Translation: First do no harm)-The effects of sodium hypochlorite on dentin as used in endodontics’ [7]. The authors stated that little is known about the deleterious effect of SH on dentine, such as post-treatment root fracture causing tooth loss. This study used transmission electron microscopy, and revealed collagen destruction on the surface and subsurface of dentine that had been treated with high concentrations of SH for long contact times. Size exclusion chromatography showed that the hypochlorite anion, because of its small size, penetrated the water compartments of apatite-encapsulated collagen fibrils. These fibrils were degraded, causing a 25–35 μm thick, non-uniform “ghost mineral layer” to develop, which had enlarged coalesced dentinal tubules and lateral branches [7]. A major limitation was that the study assessed the effects of 8% SH in contact with dentine for up to 4 h. These parameters are not consistent with the use of SH in clinical endodontic practice. SH is typically used in clinical practice at concentrations from 0.5% to 6%, although a product with a concentration of 8.25% became available in 2012 [7]. From a clinical perspective, SH provides good anti-bacterial properties when used within the range of 1–5.25% [8]. However stronger concentrations of SH are more effective for dissolving tissue and also for causing collagen deproteination [5]. In light of these and other studies on the effects of SH on the mechanical properties of dentine, a further review of the evidence is warranted.

Calcium hydroxide (CH) has a highly alkaline nature due to release of hydroxyl ions. Released ions are responsible for antimicrobial actions [9,10,11] and for promoting mineralized tissue formation [12]. CH has been used as an intra-canal medicament between appointments [13], as well as for apexification [14,15], apexogenesis [15], arrest of root resorption defects [9,16], and direct and indirect pulp-capping procedures [9,17]. CH materials have also been used for retrograde root fillings and as root canal sealers. However, since most CH hydroxide materials are soluble and can dissolve in the presence of tissue fluids, they have not been a permanent solution for some of these clinical applications. When used for apexification, CH pastes require frequent replacement over a 6–18 month period to achieve dentinal bridge formation [12]. 

The high alkalinity of CH has led to investigations of the effects of CH on root dentine over the long term. Several in vitro studies have found an association between deteriorations in strength and other physical properties [18,19,20,21,22,23,24,25,26,27,28,29,30]. However, recently published studies found no significant differences in fracture strength between sheep incisor roots filled with calcium hydroxide and untreated control groups at the 2-month time point [31] and over a 6-month period [32]. A further recent study has corroborated these findings by showing no significant differences in the fracture strength of CH treated and untreated teeth over a 9-month period in vitro [33]. 

MTA has been advocated for several of the same clinical procedures as CH [34,35,36,37]. MTA has the added advantage of providing a good seal at the location where it is placed, preventing microleakage [38]. MTA promotes the formation of mineralized tissue when it is placed in contact with dental pulp or peri-radicular tissues [37]. It can also promote pulpal regeneration in immature permanent teeth that have sustained an insult from dental caries or trauma [39]. Several studies have found that MTA, when used in a similar manner to CH, has (a) no effect on the fracture resistance or strength of roots [21,40], or (b) that MTA-treated roots displayed superior fracture strength compared to CH-treated roots [26]. Other studies have reported that physical properties such as the flexural strength of root dentine reduced significantly when treated with MTA for 3 months [27] and for 12 months [41] when compared to untreated control samples. 

The aim of this review was to identify and analyze studies related to the effects of alkaline materials used in dentistry on root fracture resistance and other relevant mechanical properties. 

## 2. Search Strategy and Criteria for Inclusion

A PubMed search was conducted for published articles in the English language, between 1982 and 2017. The search terms for SH as an irrigating solution were the same MeSH terms as employed by Pascon et al. [6] in their 2009 review. The inclusion criterion for SH was that studies investigated the direct effect of SH on the mechanical properties of dentine.

**Figure 1:**
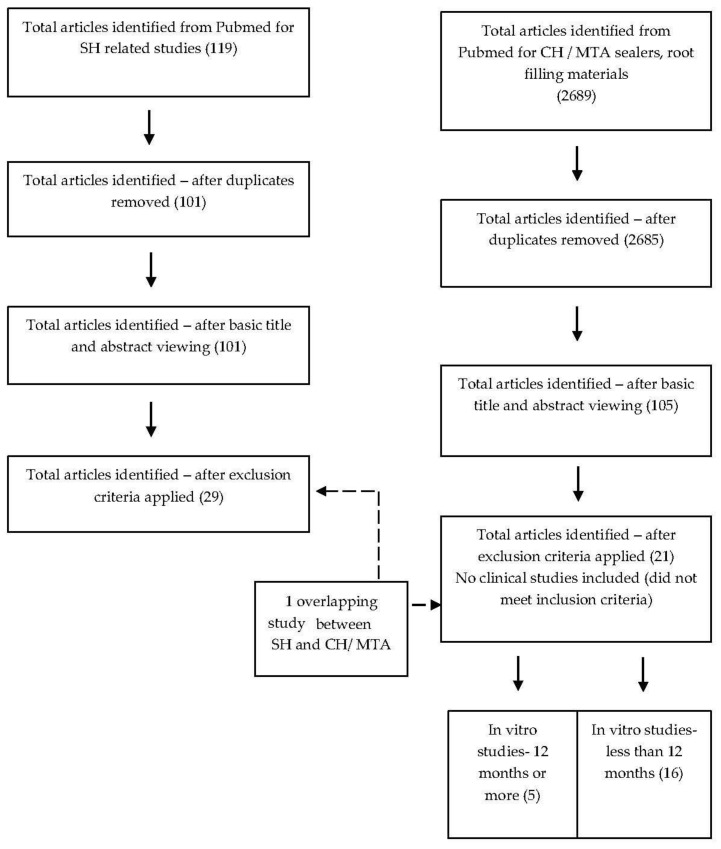
Flow diagram indicating the search strategies for the review on alkaline materials and effect on root dentine. Legend: SH—Sodium hypochlorite, CH—Calcium hydroxide, MTA—Mineral Trioxide Aggregate.

**Figure 2:**
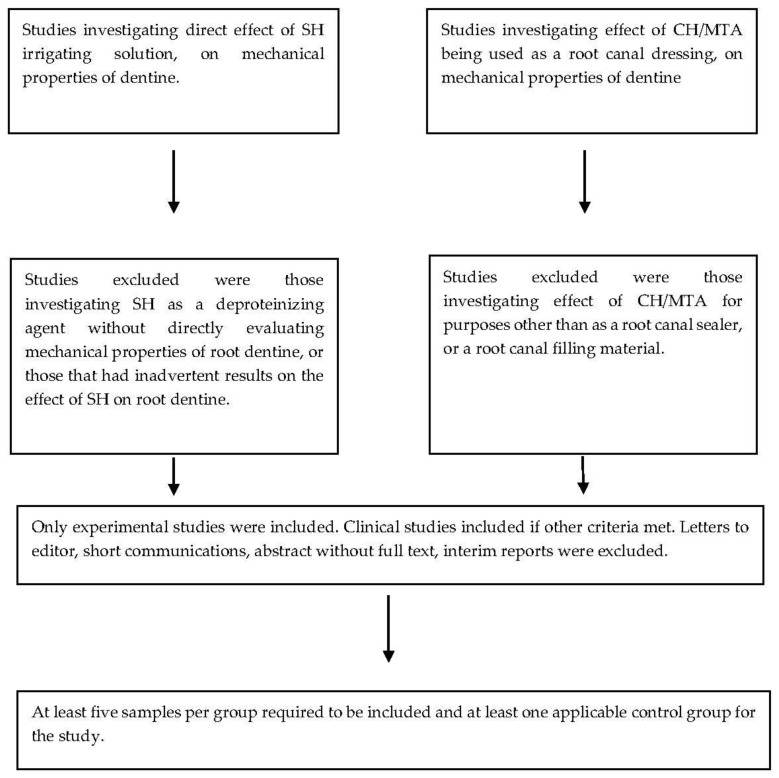
Flow diagram indicating inclusion criteria for the current review. Legend: SH—Sodium hypochlorite, CH—Calcium hydroxide, MTA—Mineral Trioxide Aggregate.

For CH/MTA-related materials, the search terms included “alkaline material”, “calcium hydroxide” “mineral trioxide aggregate” and “dentine” or “root canal dentine” combined with MeSH terms such as “compressive strength”, “elastic modulus” “flexural strength” or “fracture strength” or “fracture resistance”. The search was enhanced by hand searching relevant references from the reference list for each article. To be included, the article was required to have at least one experimental group with tooth roots filled with or treated with some form of alkaline material such as calcium hydroxide or mineral trioxide aggregate; to have at least one applicable control group; and a minimum of 5 samples per experimental group. Only full experimental studies were included. Letters to the editor, short communications, abstracts, interim reports and case reports were excluded. The flow diagram for the search strategy for both aspects is detailed in Figure 1 and inclusion criteria are described in Figure 2. 

Data extracted from the included articles was recorded on a standardized form, and included author and year of publication, type and number of teeth in the study, length of time that the roots were exposed to the alkaline material, type of mechanical property tested and any other relevant outcomes related to this review. Information was also recorded on the concentration of sodium hypochlorite and, for CH/MTA studies, the type of alkaline material used as a dressing or root filling was noted. 

## 3. Results

As shown in Figure 1, after removal of duplicates, screening of abstracts and application of inclusion criteria, 29 articles were identified for SH and 21 articles for CH and MTA. There was one included study that overlapped criteria for SH and CH/MTA. 

Table 1, Table 2, Table 3, Table 4 and Table 5 show the main findings and descriptions of the included studies for SH. The SH solutions tested had concentrations ranging 0.5–8.25%, with contact times ranging from 1 min to 4 h.

For SH investigations, most of the studies that tested flexural strength, elastic modulus and compressive strength used rectangular-shaped dentine bars, with the 3-point bend test. The studies that examined roughness employed intact teeth where the crown and/or root was sectioned and tested for either Knoop hardness or Vickers hardness. Nearly all studies looking at the effects of SH tested human permanent teeth, while one study assessed deciduous teeth [42] and only two studies tested bovine dentine [43,44]. 

Table 6, Table 7, Table 8 and Table 9 show the main findings and description of included studies for CH/MTA and related materials.

Of the studies that investigated the effect of CH/MTA and similar products, four studies each used immature lamb/sheep incisors and bovine teeth, while the remaining 13 studies used human permanent teeth. The CH/MTA studies tested CH powder and MTA mixed with different vehicles, proprietary products containing different concentrations and vehicles for CH, MTA including white MTA, Biodentine, CEM and other calcium silicate-based root canal sealers. A number of evaluation methods were employed. 

Eleven of the 21 studies [19,22,23,24,27,28,29,30,31,32,45] tested the effect of alkaline materials on roots of teeth or radicular dentine for 12–24 months (5 studies) or 12 months and less (six studies) after sample preparation [22,23,24,27,32,45]. Seven of the remaining 10 studies, tested effects at a pre-determined single time point either soon after the samples were prepared or within a week [18,40,46,47] or at 30 days [25,26,48]. The last three studies tested at a single time point between 100 days and 12 months [21,41] and one study investigated effects at two time points (30 days and 180 days) [20]. 

Four of the five studies that looked at effects over 12–24 months showed a significant reduction in mechanical properties for root dentine in immature sheep incisors [19], human teeth [28,29] and bovine teeth [30] that had been treated with CH, while only one study showed no significant differences of mechanical properties of immature sheep incisors that were treated with MTA and CH compared with untreated samples [31]. 

Three of the six CH/MTA studies that explored effects up to 6 months or less, showed a significant drop in mechanical properties such as: (a) reduced microtensile fracture strength in human incisor roots treated with CH tested at multiple time points up to 84 days [22]; (b) lower flexural strength when human third molars were treated with suspensions of nanometric bioactive glass 45S5 for up to 30 days [24]; (c) reduced modulus of toughness and flexural strength of human third molar root dentine when Biodentine and MTA-plus were tested for up to 3 months. There were no significant differences when human anterior teeth roots were treated with MTA and subjected to vertical fracture [45], or when immature lamb incisors were treated with three different CH formulations for up to 6 months [32]. There was a significant increase in elastic moduli when bovine incisors were treated with a CH paste compared to a control [23]. 

A recent study that investigated three different CH formulations over 9 months found no significant reduction in fracture resistance of immature lamb incisors when compared to a saline control group [33]. 

With short-term effect studies involving CH/MTA-like alkaline materials, human maxillary incisors treated with calcium silicate-based sealers performed either much better than the control groups [40] or had no significant differences from control [47]. When the effects of CH/MTA like materials were investigated for up to a week, it was seen that (a) when human teeth were treated with CH powder mixed with distilled water, flexural strength reduced significantly but not the modulus of elasticity [18]; (b) treatment of human maxillary incisors with MTA, Biodentine and CEM did not reduce fracture strength [46]. 

A decrease in the load required for failure of human premolars treated with CH paste was noted for an investigation over a 30-day period, with a single test point [25]. There was also a reduction in flexural strength in bovine incisors when treated with CH, MTA and CEM [26], as well as a reduction in fracture strength when human incisors were treated with MTA [48].

A study that investigated the effect of a CH formulation, MTA and a combination of the two on immature sheep incisor roots over 100 days found a significant reduction in fracture resistance when CH was used by itself [21]. Another study examined the effect of a CH slurry with a proprietary CH product on human teeth, and found a significant reduction (19%) at 180 days but no difference at 30 days compared to controls. [20]. Biodentine and white MTA powder when mixed with distilled water and placed in contact with human maxillary anterior teeth produced a significant decrease in fracture resistance over 12 months [41].

## 4. Discussion

Pascon et al. [6] in their 2009 review of the deleterious effects of SH on dentine identified 55 papers of which 16 met their inclusion criteria. Using the same search terms, in the present study 101 papers were identified, with 29 papers meeting the inclusion criterion. Overall, a number of studies have demonstrated degradation of the root and weakening of dentine with extended time of contact and with greater SH concentration [7,49,50]. The higher concentration of 8.25% that has become commercially available since 2012 [7] is of note because this causes considerable degradation of dentine to a depth of 25–35 μm after 4 h. This contact time is far longer than most endodontic procedures, especially with the advent of rotary nickel–titanium instrumentation which decreases the time required for chemo-mechanical preparation. Using a more realistic exposure time of 60 min, Cullen in 2015 showed that 8.25% SH had no significant effect on the flexural strength or the elastic modulus of dentine [51]. 

Whilst the majority of studies have reported a degradation of the mechanical properties of roots caused by SH, some investigations that have used similar methodologies failed to find significant effects [5,51,52,53]. Further studies are required to analyze this anomaly. Any deleterious effects of SH are a clinical concern due to the potential for root fracture, which could led to tooth loss. The majority of studies indicate that higher SH concentrations cause greater hard-tissue degradation. Because SH is bactericidal at concentrations of only 1%, from a clinical perspective it would seem prudent to use this low concentration of SH in cases where the canal is pulpless, and for retreatment cases where the pulp has been previously removed, since in both cases the tissue-dissolving capabilities of SH are not required. On the other hand, higher concentrations of SH may be beneficial when pulpitis is the initiator of endodontic treatment, as dissolution of organic pulp tissue will be required. 

With regard to calcium hydroxide’s effects on root strength with extended exposure times, it is known that when traumatized necrotic immature permanent anterior teeth are treated, a range of factors affect the success rates for inducing a calcific barrier and completing root formation [54]. The reported times required for a calcific barrier are between 5.1 and 6.8 months [55,56] for calcium hydroxide, with 2–3 dressings over that time [55]. Follow-up times of 12 months or more are recommended after calcific bridge formation has occurred, since one form of long-term failure is root fracture [54]. Two retrospective clinical studies investigated root fractures of immature teeth after apical barrier formation and obturation, using follow-up periods longer than 12 months [57,58]. These were not included in this review due to a lack of a suitable control group for comparison. They reported a 40% and 32% incidence of fracture, respectively, in teeth that had been subjected to long-term treatment to CH intracanal dressing after trauma. However, teeth with completed root development demonstrated only a 2% incidence of cervical fracture as opposed to a 77% incidence of cervical fracture where root development was less than 50% complete. Additionally, only 34% of teeth without a healed cervical resorptive defect had fractured. 

An analysis of long-term complications after apexification [59] showed that for cervical root fractures, secondary trauma was a confounding factor in 85% of cases. Other factors such as type of restoration, technique of apexification, stage of root development or the presence of cervical resorptive defects were not identified. 

The present review included only five in vitro studies that investigated the effects of CH/MTA over 12 months or more. Four of these showed a significant reduction in mechanical properties, when immature sheep incisors [19], human teeth [28,29] and bovine teeth [30] were treated with different formulations of CH. However, two of these studies had no control group for the test time points [19,28] and the baseline value for the control group in one study [19] was not determined experimentally within the same study, but rather was taken from a previous study. Additionally, the effects of storage media on mechanical properties were not investigated. Fracture resistance was recorded as the mean compressive force to failure in one study [29]. With anatomic samples, the lack of homogeneity in shape and size of the teeth and roots is an important factor to consider. This inherent variation is usually compensated for in strength studies by factoring in the true cross-sectional area of the fractured sample. It was not clear how these anatomic variations were taken into consideration for studies of human and bovine teeth [28,29,30]. It is important to note that ovine and bovine teeth have different dimensions and cross-sectional profiles than human teeth [60]. Only one investigation [31] tested the effects of multiple types of alkaline materials at multiple time points (2 weeks, 2 months and 2 years) and had a valid untreated and un-instrumented control group for the length of the study. The fracture strengths reported were higher in the treatment groups at 2 months and 2 years, and there was no significant difference between the CH and control groups. Although there was a reduction in strength in the CH groups (by 20% at one year), this was not significant when compared to the control untreated groups, which reduced by 44% at 2 months and additionally by a further 3% by 1 year. Because MTA-treated samples had a cumulative reduction in strength over 1 year by only 3%, they were stronger at 1 year than the controls. This emphasizes the need to include controls for the entire period of a study, rather than use baseline values. It is also important to compare the variations in strength within each test group over a period of time.

In terms of long term effects, the formation of a calcific bridge requires approximately 7 months with both CH and MTA-based materials [54]. A total of 16 studies that investigated the effect of alkaline materials on radicular dentine over 12 months or less were included in the present review. Of these, three studies were 6 months or less in duration, and showed a reduction in mechanical properties [22,24], and a reduction of modulus of toughness and flexural strength of human third molar root dentine when exposed to Biodentine and MTA-plus for up to 3 months [27]. However, MTA-treated groups subjected to vertical fracture showed superior results at 6 months to controls [45]. Likewise, in one study the fracture strength did not reduce significantly after three different CH formulations had been used for up to 6 months [32]. One study even showed significant increases in elastic moduli when bovine incisors were treated with a CH paste between 1 and 7 days after treatment, with no significant reduction after 90 days compared to controls [23]. An increase in the modulus of elasticity may, however, accompany reduced fracture strength. A recent study also found no difference in fracture strength values between immature lamb incisors treated with three different CH formulations and a control group in saline, tested over 9 months [33]. 

A dramatic reduction in mechanical properties over a test period of 10 days or less was an interesting outcome of this review. Dentine bars were soaked in an aqueous CH solution [18], allowing CH to contact all parts of the bars, including both internal and external root surfaces. This unrealistic contact may explain the reduction in flexural strength seen in this group after just one week. Another study immersed the prepared bars in suspensions of CH [24] after removing the outer dentine and cementum, rather than placing the materials in contact with internal root surfaces [19,21,32]. This difference in methodology may explain why there was a 30% drop in flexural strength in the CH-treated groups after 10 days compared to the saline control [24]. Dentine samples were immersed in a 1 mm-deep layer of CH paste to result in a reduced elastic modulus after 7 days [23]. Together, these results show how exposure of dentine samples to the test solution can influence the outcomes and that variations in methods are an important consideration. None of these studies that had a dramatic reduction in properties over 10 days or less replicated the real-life application of the materials to radicular dentine in situ.

Of the studies included for CH/MTA materials, several explored fracture strength, resistance, micro-tensile fracture strength and load to failure as measures of weakening effects on root dentine [19,20,21,22,24,26,28,29,30,31,32,40,41,46,47,48]. There was a disparity in study design, making direct comparisons difficult due to variations in the types and proportions of samples, the directions of forces applied, and in the calculations for strength. Some studies measured fracture strength in MPa after taking into account both the load at failure and the specific cross-sectional area of the fractured samples [19,21,22,31,32,41], while others simply recorded the maximum force at failure and reported this value as the fracture strength or resistance [20,28,29,40]. 

Any claimed reduction in mechanical properties of roots caused by CH formulations has been attributed to its highly alkaline pH, which is thought to denature the dentine collagen network or links between collagen and hydroxyapatite crystals [19,20,28]. It has been suggested that increased expression of specific matrix metalloproteases (MMP-2, MMP-14) could contribute to the degradation of type I collagen in dentine [31,32] and is, therefore, attributed to an increased risk of root fractures. However, other authors [32,33] find that CH may not weaken teeth when used long term and hence the effect of increased expression of specific matrix metalloproteases is unclear. These links proposed previously for matrix metalloproteases are also counter to the data for MTA and similar tricalcium silicate materials (TCSM) such as Biodentine, which generate calcium hydroxide and have a highly alkaline nature. Such materials appear to cause an increase in mechanical properties. This strengthening effect has been attributed to a hydroxyapatite-like layer that forms between dentine and TCSMs through the hydration reaction of TCSMs in the presence of tissue fluids [41,45]. Furthermore, MTA induces expression of a tissue inhibitor of metalloproteinase (TIMP-2) in the dentine matrix, which then prevents destruction of the collagen matrix [31,32]. 

Results from in vitro studies cannot be translated directly to clinical conditions. This is especially important to note when considering a human immature tooth that is being treated for a procedure requiring the long-term application of a material such as CH or MTA. The functioning tooth is supported by a highly specialized periodontium, which undergoes elastic deformation when subjected to load [54] and this is hard to replicate in vitro. The teeth used in laboratory tests were subjected to mechanical forces while embedded in rigid materials such as plaster of Paris or acrylic resin, which do not behave in the same manner as the specialized periodontium. 

## 5. Conclusions

This review has identified some studies that suggest that alkaline materials may degrade the strength of dentine, while other studies have shown little deleterious effects. As the effects of SH are time- and concentration-dependent, it is advisable to use higher concentrations of SH when pulp tissue is present in the canal as determined by a diagnosis of pulpitis. For teeth with a diagnosis of necrosis, and for pulpless or previously root filled teeth, there is no requirement for the tissue-dissolving capabilities of SH. In terms of the use of CH and MTA, the long-term dressing of immature teeth does not weaken teeth, since more recent and better designed studies show no weakening of teeth over time, unlike what was seen in earlier in vitro studies which had experimental design problems. This highlights the difficulty of translating the findings of in vitro studies into clinical practice.

## Figures and Tables

**Table 1:** MeSH search for Dentine OR Root canal dentine AND (Sodium hypochlorite) AND Compressive Strength.

First Author/Year	NaOCl Irrigating Solution Concentration Used	Time	Evaluation Methods	Result
Sim 2001	0.5% and 5.25%	2 h	EMT/FST/CLT	Concentration-dependent reduction in compressive strength.
Hillesheim 2017	1%	40 min	CLT	Significant reduction in compressive strength with 17% EDTA, 15 NaOCl and 30 days Ca(OH)_2._
Cecchin 2017	6%	30 min	CLT	NaOCl significantly reduced compressive strength.

Legend: NaOCl—Sodium hypochlorite; CLT—Compressive load test; FST—Flexural strength test; CyLT—Cyclic load test, EMT—Elastic modulus test.

**Table 2:** MeSH search for Dentine OR Root canal dentine AND (Sodium hypochlorite) AND Elastic Modulus.

First Author/Year	NaOCl Irrigating Solution Concentration Used	Time	Evaluation Methods	Result
Grigoratos 2001	3% and 5%	2 h	EMT/FST	Reduction in elastic modulus for NaOCl.
Sim 2001	0.5% and 5.25%	2 h	EMT/FST/CLT	Reduction in elastic modulus.
Marending 2007	2.5%	24 min	EMT/FST	Elastic modulus unaffected.
Jungbluth 2011	5%	30 min	EMT/FST	Reduction in elastic modulus.
John 2013	5%	36 min	EMT	Reduction in elastic modulus.

Legend: NaOCl—Sodium hypochlorite; EMT—Elastic modulus test; FST—Flexural strength test; CyLT—Cyclic load test.

**Table 3:** MeSH search for Dentine OR Root canal dentine AND (Sodium hypochlorite) AND Flexural strength.

First Author/Year	NaOCl Irrigating Solution Concentration USED	Time	Evaluation Methods	Result
Grigoratos 2001	3.5% and 5%	2 h	EMT/FST	Reduction in flexural strength for NaOCl group.
Sim 2001	0.5% and 5.25%	2 h	EMT/FST/CLT	Reduction in flexural strength.
Mai 2010	5.25%	10 min and 1 h	EMT/FST/TEM	1 h and not 10 min significantly reduced flexural strength.
Zhang 2010	1.3% and 5.25%	10 min to 4 h	FST/FT-IR	Decrease in flexural strength is time- and concentration-dependent.
Jungbluth 2011	5%	30 min	EMT/FST	Reduction in flexural strength.
Marcelino 2014	5.25%	5–10 min	FST/MH	No effect on flexural strength.
Souza 2014	2.5% and 5%	26 min	CLT	Reduction in flexural strength by about 30%.
Cullen 2015	8.25%	1 h	EMT/FST	NaOCl did not have a significant effect on flexural strength or modulus.
Gu 2017	2%, 4%, 6% and 8%	30 min to 4 h	FST/TEM/FT-IR	Flexural strength reductions were significant for time and concentration effects.
Cecchin 2017	6%	30 min	CLT/FTS/UTS	Significant reduction in flexural strength.

Legend: NaOCl—Sodium hypochlorite; EMT—Elastic modulus test; FST—Flexural strength test; CyLT—Cyclic load test; TEM—Transmission electron microscopy; FT-IR—Fourier transform infrared spectroscopy; MH Microhardness test; UTS—Ultimate tensile strain.

**Table 4:** MeSH search for Dentine OR Root canal dentine AND (Sodium hypochlorite) AND Hardness.

First Author/Year	NaOCl Irrigating Solution Concentration Used	Time	Evaluation Methods	Result
Slutzky-Goldberg 2002	2.5% and 6%	5, 10 and 20 min	MT (Vickers)	Microhardness decrease was time- and concentration-dependent.
Ari 2004	2.5% and 5.25%	15 min	MT (Vickers)	Microhardness significantly decreased.
Oliveira 2007	1%	15 min	MT (Vickers)	Microhardness significantly decreased.
Sayin 2007	2.5%	5 min	MT (Vickers)	Microhardness reduced.
Patil 2011	2.5% and 5%	15 min	MT (Vickers)	Microhardness reduced.
Zaparolli 2012	1%	10 min	MT (Knoop)	Microhardness reduced.
Garcia 2013	1%, 2% and 5%	15 min	MT (Knoop)	Microhardness reduced.
Pascon 2014	1%	30 min	MT (Vickers)/SEM	Microhardness reduced.
Aslaantas 2014	6%	5 min	MT (Vickers)	Microhardness significantly reduced.
Marcelino 2014	5.25%	5–10 min	MT (Knoop)	Microhardness reduced.
Ballal 2015	2.5%	1 min	MT (Knoop)	Microhardness reduced.
Saha 2017	3%	15 min	MT (Vickers)	Negligible effect on microhardness.

Legend: NaOCl—Sodium hypochlorite; MT—Microhardness test; SEM—Scanning electron microscopy.

**Table 5:** MeSH search for Dentine OR Root canal dentine AND (Sodium hypochlorite) AND Roughness.

First Author/Year	NaOCl Irrigating Solution Concentration Used	Time	Evaluation Methods	Result
Ari 2004	2.5%	15 min	MT (Vickers)	Increased roughness.
Hu 2010	5.25%	10 min	AFM	Increased roughness.
Patil 2011	2.5% and 5%	15 min	MT	Increased roughness.
Tatari 2013	2.5% and 5%	2.5–30 min	MT (Profilometer)/SRT	No effect on roughness.
Pascon 2014	1%	30 min	MT/SEM	Small change in roughness.
Oliviera 2014	1%, 2.5% and 5%	35 min	CLSM	5% significantly increased roughness.
Ballal 2015	2.5%	1 min	MT/SRT/AFM	Maleic acid increased roughness more than NaOCl.
Farshad 2017	5.25%	10 min	AFM	Significantly increased roughness.

Legend: NaOCl—Sodium hypochlorite; MT—Microhardness test; AFM—Atomic force microscopy; CLSM—Confocal laser scanning microscopy; SEM Scanning electron microscopy; SRT—Surface roughness testing.

**Table 6:** MeSH search for Dentine OR Root canal dentine AND (Calcium hydroxide OR mineral trioxide or alkaline material) AND Compressive Strength.

First Author/Year	Material Used as Dressing or Root Filling	Time	Evaluation Methods	Result
Sahebi 2010	Ca(OH)_2_	30 days	CLT	Significant reduction in compressive strength by 14.4%
EL-Ma’aita 2014	White MTA, MTA	48 h, 1, 6 months	VT	No reduction in resistance to vertical fracture load.

Legend: Ca(OH)_2_—Calcium hydroxide, MTA—Mineral Tri oxide Aggregate, CLT—Compressive load test; VT—Vertical fracture.

**Table 7:** MeSH search for Dentine OR Root canal dentine AND (Calcium hydroxide OR mineral trioxide or alkaline material) AND Elastic Modulus.

First Author/Year	Material Used as Dressing or Root Filling	Time	Evaluation Methods	Result
Grigoratos 2001	Saturated Ca(OH)_2_ solution	2 h	EMT/FST	No reduction for Ca(OH)_2_; sequential use of both had no additional weakening effect.
Kawamoto, R 2008	Ca(OH)_2_ paste	1, 7, 14, 21, 28, 56 and 90 days	EMT	Significant increases in the elastic moduli for 7 days Ca(OH)_2_ to root dentine and for 1 day to coronal dentine.
Marending 2009	Suspensions of nanometric bioactive glass 45S5; Ca(OH)_2_	1, 10 and 30 days	EMT/FST	No reduction in elastic modulus.
Sawyer 2012	Biodentine, MTA Plus	24 h, 1, 2, 3 months	FM, FST, MOT	Reduction in modulus of toughness; no significant difference for flexural modulus.

Legend: Ca(OH)_2_—Calcium hydroxide, MTA—Mineral Trioxide Aggregate, Elastic modulus test; FST—Flexural strength test; FM—Flexural modulus; MOT—Modulus of toughness.

**Table 8:** MeSH search for Dentine OR Root canal dentine AND (Calcium hydroxide OR mineral trioxide or alkaline material) AND Flexural strength.

First Author/Year	Material Used as Dressing or Root Filling	Time	Evaluation Methods	Result
Grigoratos 2001	Saturated Ca(OH)_2_ solution	2 h	EMT/FST	Saturated Ca(OH)_2_ reduced the flexural strength of dentine but not the modulus of elasticity. Sequential use of NaOCl and Ca(OH)_2_ has no additional weakening effect.
Marending 2009	Suspension nanometric bioactive glass 45S5; Ca(OH)_2_	1, 10 and 30 days	EMT/FST	Reduction in flexural strength in both groups.
Sahebi 2012	Ca(OH)_2_, MTA and CEM	30 days	FF	Reduction in flexural force to fracture.
Sawyer 2012	Biodentine, MTA	24 h, 1, 2& 3 months	FM, FST, MOT	Reduction in flexural strength.

Legend: Ca(OH)_2_—Calcium hydroxide, MTA—Mineral Tri oxide Aggregate, CEM—Calcium Enriched Mixture, EMT—Elastic modulus test; FST—Flexural strength test; TEM-Transmission electron microscopy; FF-Flexural force; FM—Flexural modulus; MOT—Modulus of Toughness.

**Table 9:** MeSH search for Dentine OR Root canal dentine AND (Calcium hydroxide OR mineral trioxide or alkaline material) AND Fracture strength OR Fracture resistance.

First Author/Year	Material Used as Dressing or Root Filling	Time	Evaluation Methods	Result
Andreasen 2002	Ca(OH)_2_	14, 30, 60, 90, 180, 270, 360 days	FS	Reduction in fracture strength.
Doyon 2005	Ca(OH)_2_	20 and 180 days	PF	No control; reduction in peak load at fracture.
Andreasen 2006	Ca(OH)_2_; MTA and combination	100 days; 70 + 30 days for combination	FS	Reduction in fracture strength when only Ca(OH)_2_ used. Use of MTA or combination had no effect.
Rosenberg 2007	Ca(OH)_2_	7, 28 and 84 days	MTFS	Reduction in micro tensile fracture strength.
Hatibovic’-Kofman 2008	Ca(OH)_2_; MTA	2 weeks, 2 months and 2 years	FS	No reduction in fracture strength at 2 years.
Sagsen 2012	Calcium silicate based sealer	Immediate	FS	No reduction in fracture strength.
Batur 2013	Ca(OH)_2_ dressing and obturated later	30, 90, 180, 270, 360, and 540 days.	MTFS	Reduction in micro tensile fracture strength.
Zarei 2013	Ca(OH)_2_	1 week, 1, 3, 6, 12 months	FS	Reduction in fracture strength.
Hawkins 2015	3 Ca(OH)_2_ formulations	1, 3, 6 months	FS	No reduction in fracture strength.
Elnaghy 2015	Biodentine, white MTA	12 months	FR	No significant difference between experimental groups, but positive control had highest resistance to fracture.
Valera 2015	Ca(OH)_2_	15, 60, 90, 180, and 360 days	FR	Reduction in fracture resistance with NaOCl irrigation groups.
Evren 2016	MTA, Biodentine, and CEM	1 week	FS	No reduction in fracture strength.
Karapinar-Kazandag et al. 2016	MTA compared to flowable and hybrid composite for reinforcing effect	30 days; control only for 1 week	FR	Significant difference between immature unfilled, instrumented roots and mature root segments. No difference in fracture strength between groups mature untreated root segments, immature treated roots filled with either MTA, flowable composite or hybrid composite groups.
Aksel 2017	MTA with 3 vehicles	Immediate	FR	No reduction in fracture resistance with MTA groups compared to control.
Kahler 2017 In press	3 Ca(OH)_2_ formulations	0, 3, 6, 9 months	FS	No reduction in fracture strength compared to saline controls over 9 months.

Legend: Ca(OH)_2_—Calcium hydroxide, MTA—Mineral Tri oxide Aggregate, CEM—Calcium Enriched Mixture, FS—Fracture strength; FR—Fracture resistance; CyLT—Cyclic load test; PF—Peak load at fracture; MTFS—Micro tensile fracture strength.
